# Patient safety culture among the nursing staff and quality assurance managers at Gauteng public hospitals

**DOI:** 10.4102/hsag.v30i0.3136

**Published:** 2025-10-28

**Authors:** Lowani R. Serongwa, Kholofelo L. Matlhaba

**Affiliations:** 1 Department of Health Studies, College of Human Sciences, University of South Africa, Pretoria, South Africa

**Keywords:** nursing staff, patient safety, patient safety culture, public hospital, quality assurance manager

## Abstract

**Background:**

Determining the level of patient safety culture is important in identifying areas for improvement in patient safety and care. The desire to improve patient care motivated this study.

**Aim:**

The aim of this study is to determine the level of patient safety culture among the nursing staff and quality assurance managers at the three selected public hospitals in Gauteng province, South Africa.

**Setting:**

The research was conducted at the three selected public hospitals in Gauteng province, South Africa, categorised as central, regional and district.

**Methods:**

Descriptive quantitative method had two sub-phases. Phase 2(a): a questionnaire administered to professional nurses, enrolled nurses and enrolled nursing assistants. Phase 2(b): a questionnaire administered to operational managers of nursing, assistant managers of nursing and quality assurance managers. Simple random sampling yielded a response rate of 87.2% (436/500).

**Results:**

Three dimensions – supervisor, manager or clinical leader support for patient safety; handoffs and information exchange and teamwork – had the highest average positive response rates. Staffing and work pace and reporting patient safety incidents had moderate average positive response rates. Five dimensions had low average positive rates: organisational learning – continuous improvement, communication openness, hospital management support for patient safety, response to error and overall perceptions of patient safety. The overall patient safety culture scored 51.26%, indicating a moderate average positive response rate.

**Conclusion:**

The level of patient safety culture needs improvement.

**Contribution:**

This study contributes to the comprehension of patient safety culture within public hospitals and provides healthcare leaders with improvement areas.

## Introduction

Ensuring patient safety is an integral aspect of providing quality healthcare services. It involves systematically minimising risks and preventing avoidable harm to patients during the provision of care. A robust patient safety culture supports improved health outcomes by embedding safety-focused values, practices and leadership within healthcare systems. According to Harbi et al. (2022), ‘patient safety culture refers to the recognised beliefs, norms, values, appropriate behaviour and suitable attitudes within a workplace environment’.

Patients suffer harm in healthcare facilities and die unnecessarily across the world (World Health Organization 2020). In fact, the World Health Organization (2020) reports that up to 40% of patients globally may experience harm in primary or outpatient care settings during the course of treatment.

There are different tools utilised to gather data on patient safety culture, namely the Hospital Survey on Patient Safety and Culture (HSOPSC) tool developed by the Agency for Healthcare Research and Quality (AHRQ) at Rockville, United States (Famolaro et al. 2016). The tool comprises the composites of patient safety culture, which include communication openness, feedback and communication about error, handoffs and transitions, management support for patient safety, nonpunitive response to error, organisational learning – continuous improvement, overall perceptions of patient safety, staffing, supervisor and/or manager expectations and actions promoting patient safety, teamwork across units and teamwork within units (Famolaro et al. 2016). Below are some of the studies conducted by the researchers using the Hospital Survey on Patient Safety Culture tool.

From Meena and Shetty’s (2021) study, ‘dimensions of communication openness, non-punitive response to errors and handoff and transitions and staffing about patient safety had received the least positive responses’. In a similar study conducted in Nigeria by Kaware et al. (2022), it was discovered that staffing had the highest negative responses (69.5%), followed by non-punitive response to error (57.2%) and frequency of event reporting (56.9%). From the study conducted by Bongongo et al. (2023:1):

Public healthcare workers in Tshwane’s regions 1 and 2 have a good patient safety culture, and that it is particularly positive among nurses with more experience and providing services at the primary care level. (p. 1)

During monitoring and support visits on the provision of quality patient care, the researcher observed a lack of leadership commitment with adequate resources for quality patient care, a lack of commitment to improving work processes and patient outcomes and, lastly, a lack of continuous learning from the mistakes and successes in managing patient safety incidents.

Although a related study was conducted in other settings within the province, the present study was undertaken because the contexts differ in terms of hospital categorisation, package of services rendered, population characteristics and resource allocation and availability. Exploring the phenomenon in diverse settings enhances the robustness and transferability of findings, ensuring that recommendations are relevant across the province.

Based on the above-mentioned background, the aim of this study was to assess the level of patient safety culture among the nursing staff and quality assurance managers at the three selected public hospitals in Gauteng province, South Africa. Knowledge of the level of patient safety culture within the public hospitals could provide healthcare leaders with areas for improvement. By implementing the recommended strategies, healthcare establishments can cultivate a positive patient safety culture, reduce patient safety incidents and improve patient outcomes.

## Research methods and design

### Study design

According to Nwabuko et al. (2024), research design is a structured framework or methodological approach used for data collection and analysis of a research problem. The current study utilised a descriptive quantitative approach to assess the level of patient safety culture among the nursing staff and quality assurance managers at the three selected public hospitals in Gauteng province, South Africa. A descriptive quantitative approach was used to analyse a large sample size, utilising descriptive statistics like frequencies and percentages to summarise the data. Quantitative research involves the systematic investigation of measurable phenomena and typically incorporates controlled procedures and statistical analysis to interpret results (Polit & Beck 2022). In this study, the concept of patient safety culture was explored in depth to generate meaningful, evidence-based conclusions.

### Setting

Polit and Beck (2021) define natural setting as ‘an uncontrolled, real-life environment that the researcher does not manipulate or alter for the purpose of the study’. The research was conducted at the three selected public hospitals in Gauteng province, South Africa. The three selected public hospitals are categorised as one central hospital, one regional hospital and one district hospital. These facilities were chosen to ensure varied contextual representation across the provincial public healthcare system.

### Study population and sampling strategy

#### Study population

Gray and Grove (2020) state that a population is a specific type of individual or element that is the centre of the study. The study population was composed of professional nurses, enrolled nurses, enrolled nursing assistants, operational managers of nursing, assistant managers of nursing and quality assurance managers available, willing and consenting to participate in the study and meeting the set criteria.

***Sample size:*** Polit and Beck (2021) define sample size as the total count of units of analysis (such as individuals, families, groups, patients or communities) to be included in a study. The units of analysis in this study were professional nurses, enrolled nurses, enrolled nursing assistants, operational managers of nursing, assistant managers of nursing and quality assurance managers.

The sample size for this study was calculated using the Raosoft online sample size calculator (Raosoft 2022). Based on a 95% confidence level, a 5% margin of error and an estimated 50% response distribution, a minimum of 356 participants was required. To improve the likelihood of achieving this target, a total of 500 questionnaires were distributed, incorporating a 28.8% buffer (144 additional questionnaires) to maximise response rates. Of these, 436 completed questionnaires were returned, yielding a response rate of 87.2%. The respondents consisted of nursing personnel across different professional categories, as well as quality assurance managers.

#### Sampling strategy

According to Casteel and Bridier (2021), the sampling method refers to the approach employed by the researcher to acquire the members of the sample. A probability sampling approach was utilised in this descriptive quantitative study. Probability sampling denotes that every individual within the population has a predetermined chance of being selected for inclusion in the sample (Makwana et al. 2023).

Eligible participants included professional nurses, enrolled nurses, enrolled nursing assistants, operational managers of nursing, assistant managers of nursing and quality assurance managers. Participants were required to have direct or supervisory experience in managing patient safety issues within the selected public hospitals in Gauteng province and to be willing to provide informed consent. Consent forms were shared via WhatsApp and in printed format, allowing for voluntary participation in either format.

To promote fairness and avoid selection bias, simple random sampling was applied. As defined by Polit and Beck (2021), this method ensures each eligible participant has an equal and independent probability of being chosen. A list of 739 eligible candidates was compiled in collaboration with gatekeepers – namely the nursing service managers – who facilitated access and helped coordinate structured meetings with relevant staff. During these sessions, participants were briefed on the study’s purpose, procedures and ethical safeguards to ensure fully informed participation.

The random number generator was applied to the compiled eligible candidates list, which randomly selected individuals, preserving methodological rigour. This approach ensured diversity across participant categories and enhanced the representativeness and generalisability of the findings. The transparent application of this sampling technique contributed to the credibility of the study and reinforced the validity of conclusions drawn regarding patient safety culture within the selected facilities.

***Inclusion criteria:*** Inclusion criteria include the following: Operational managers of nursing, assistant managers of nursing, quality assurance managers, professional nurses, enrolled nurses and enrolled nursing assistants who had worked at the study site for a period of 1 year or more, were willing and consenting to participate in the study and were on duty during data collection.

***Exclusion criteria:*** Individuals from the aforementioned categories were excluded if they were staff members at the study settings for less than 1 year, were not scheduled to be on duty during the data collection period or declined to provide informed consent.

#### Recruitment of the participants

Participant recruitment commenced after hospital management approval was granted. A research assistant, who held a master’s degree in nursing, coordinated initial meetings with the nursing service managers to outline the study and discuss access protocols for engaging the nursing staff and quality assurance managers. The purpose and procedures of the study were clearly explained to potential participants. Those who agreed to take part provided informed consent either via Microsoft Forms or by completing hard-copy forms. To facilitate participation, the research assistant established WhatsApp groups and distributed the Microsoft Forms link through these groups.

Hospital boardrooms and auditoriums were made available for data collection sessions. In these venues, groups of participants completed the self-administered questionnaires either electronically or in printed form. The research assistant later captured the responses from the hard copies into Microsoft Forms to ensure uniform data entry and analysis.

### Data collection

According to Gray and Grove (2020), data collection is an explicit, methodical process of gathering information that aligns with the research purpose, questions or specific objectives. In this study, data were collected using a self-administered questionnaire, which Polit and Beck (2022) define as a tool designed to gather self-reported data from participants.

Sürücü and Maslakçı (2020) define reliability as the consistency and stability of the measurement equipment over time. A clearly constructed questionnaire was verified by the supervisors and pretested at the public hospital that was not part of the current study.

Validity is the precision with which the instrument measures the construct it is designed to measure (McClure 2020). A thorough literature review was done before the instrument was developed, and the instrument was pre-tested at the public hospital that was not part of the current study.

To gather data, the HSOPSC tool developed by AHRQ was adopted and customised for the local setting by changing staff positions and primary units or work areas. The instrument included 33 items grouped into 11 composites, each rated on a 5-point Likert scale. Response categories varied depending on the item, including ranges such as ‘strongly disagree’ (1) to ‘strongly agree’ (5), ‘poor’ (1) to ‘excellent’ (5) and ‘never’ (1) to ‘always’ (5). Internal consistency was assessed using Cronbach’s alpha, with a threshold of 0.7 indicating acceptable reliability (Lobiondo-Wood & Haber 2022). The calculated Cronbach’s alpha for the instrument was 0.7265, confirming adequate internal consistency in measuring patient safety culture.

The questionnaire was structured into six thematic sections, each aligned with specific composites of patient safety culture. Section A covered teamwork, staffing and work pace, organisational learning and response to error. Section B focused on leadership support for patient safety, while Section C addressed issues related to communication about errors and openness. Section D concentrated on the reporting of patient safety incidents, and Section E assessed overall perceptions of patient safety. Lastly, Section F explored hospital management’s support for patient safety, as well as the effectiveness of handoffs and information exchange. Together, these sections captured the key elements of patient safety culture and contributed to the overall score used to evaluate perceptions among nursing staff and quality assurance managers at the selected public hospitals.

Data collection was conducted in two phases to capture perspectives at different levels of nursing practice. The first phase involved administering the questionnaire to professional nurses, enrolled nurses and enrolled nursing assistants directly involved in patient care to understand their frontline experiences. The second phase targeted operational managers of nursing, assistant managers of nursing and quality assurance managers to explore leadership, policy and administrative perspectives. This phased approach allowed for a more comprehensive understanding of the phenomenon by integrating both operational and managerial viewpoints, thereby strengthening the depth and applicability of the findings. The data collection period extended from 18 July 2024 to 26 November 2024.

### Data analysis

Data analysis is the process of examining and interpreting data to draw meaning, enhance understanding and build empirical knowledge (Gray & Grove 2020).

The research assistant sent the captured data in a Microsoft Excel spreadsheet to the researcher, who then submitted the spreadsheet to the statistician for analysis. The Statistical Package for Social Sciences Version 26.0 was utilised to conduct the analysis. According to Aljohani et al. (2021), descriptive statistics is a form of mathematical approach to synthesise, summarise and describe data acquired from a sample through frequency and percentage distributions. These statistics were utilised to summarise the frequencies and percentages and discover patterns in patient safety culture at the three selected public hospitals in Gauteng province, South Africa. A composite positive response rate on patient safety culture refers to the percentage of respondents who responded positively (e.g. ‘agree’ or ‘strongly agree’) to a set of questions directly relevant to patient safety culture. Furthermore, the composite positive response rate is calculated by combining the percentage of respondents who selected ‘agree’ or ‘strongly agree’ across various questions related to patient safety culture.

Mamman, Lilian and Emmanuel (2022) refer to inferential statistics as the process of making inferences or drawing conclusions and/or generalisation regarding specific characteristics, considering the nature or characteristics of the sample. These statistics were utilised to make inferences about the patient safety culture across the three hospitals. Pearson’s Chi-square test was utilised for inferential statistics. The Pearson’s Chi-square test was employed to examine the association between two categorical variables. The findings were evaluated at a 0.05 significance level, meaning the results were considered significant if the observed *p*-value was below 0.05.

### Ethical considerations

An application for full ethical approval was made to the College of Human Resources Research Ethics Review Committee of the University of South Africa and ethics consent was received on 8 May 2024. The ethics approval number is 41324722_CREC_CHS_2024. In addition, formal permission was granted by the management of the three selected public hospitals in Gauteng Province, South Africa.

The study was conducted in accordance with the ethical principles of respect for persons, beneficence and justice, as outlined by Botma et al. (2022). To uphold respect for persons, participants were provided with detailed information about the study and given the opportunity to voluntarily sign informed consent forms. They were also assured of their right to withdraw from the study at any point without fear of negative consequences.

To ensure beneficence, measures were taken to safeguard participants from harm or discomfort. Data collection occurred in designated hospital boardrooms, offering a safe and private environment. Additionally, participants had the option to complete the questionnaire at a time and place that suited them.

The principle of justice was observed by selecting participants strictly according to the study’s inclusion criteria, ensuring fair and equitable participation. Confidentiality was maintained throughout the research process, with access to the collected data restricted solely to the research team.

## Results

The results were presented under the headings: demographics of the respondents, comparable findings for the professional nurses, enrolled nurses and enrolled nursing assistants and operational managers of nursing, assistant managers of nursing and quality assurance managers on the composites of patient safety culture and the comparable findings for the three hospitals on the composites of patient safety culture.

Table 1 presents the demographics of the respondents.

**TABLE 1:** Demographics of the respondents.

Position	Number of the respondents	Percentages
Assistant manager of nursing	28	6.4
Operational manager of nursing	50	11.5
Quality assurance manager	9	2.1
Professional nurse	181	41.5
Enrolled nurse	96	22.0
Enrolled nursing assistant	72	16.5

**Total**	**436**	**100.0**

*Source*: Adapted from: Famolaro, T., Yount, N.D., Burns, W., Flashner, E., Liu, H. & Sorra, J., 2016, *Hospital survey on patient safety culture: 2016 user comparative database report*, AHRQ Publication No. 16-0021-EF, Agency for Healthcare Research and Quality, Rockville, MD

Table 1 shows that the majority of respondents were professional nurses, accounting for 181 out of 436 participants (41.5%). This was followed by enrolled nurses (96 respondents; 22.0%) and enrolled nursing assistants (72 respondents; 16.5%). Operational managers of nursing constituted 50 respondents (11.5%), while 28 participants (6.4%) were assistant managers of nursing. The smallest group comprised quality assurance managers, with only 9 respondents (2.1%) participating in the study.

The next table presents comparative findings between the professional nurses, enrolled nurses and enrolled nursing assistants and operational managers of nursing, assistant managers of nursing and quality assurance managers across the various composites of patient safety culture.

Table 2 presents a comparison of composite scores on patient safety culture between professional nurses, enrolled nurses and enrolled nursing assistants and operational managers of nursing, assistant managers of nursing and quality assurance managers. Both groups reported high average positive response rates for the composites ‘Supervisor, manager or clinical leader support for patient safety’ and ‘Handoffs and information exchange’, ranging from 73.56% to 90.73%.

**TABLE 2:** Comparable findings for the professional nurses, enrolled nurses and enrolled nursing assistants, and operational managers of nursing, assistant managers of nursing and quality assurance managers on the composites of patient safety culture.

Composite	Percentage	
	Professional nurses, enrolled nurses and enrolled nursing assistants composite positive response rate (*N* = 349)	Operational managers of nursing, assistant managers of nursing and quality assurance managers composite positive response rate (*N* = 87)
1. Supervisor, manager or clinical leader support for patient safety	90.73	77.78
2. Handoffs and information exchange	86.44	73.56
3. Teamwork	85.48	62.84
4. Communication about error	80.32	19.92
5. Staffing and work pace	60.03	44.83
6. Communication openness	55.37	4.89
7. Organisational learning – continuous improvement	43.75	57.86
8. Hospital management support for patient safety	30.37	32.18
9. Response to error	27.15	24.71
10. Overall perceptions of patient safety	18.34	25.29
11. Reporting patient safety incidents	10.17	17.24
**Overall level of patient safety culture**	**53.47**	**40.10**

*Source*: Adapted from: Famolaro, T., Yount, N.D., Burns, W., Flashner, E., Liu, H. & Sorra, J., 2016, *Hospital survey on patient safety culture: 2016 user comparative database report*, AHRQ Publication No. 16-0021-EF, Agency for Healthcare Research and Quality, Rockville, MD

Notes: ***N*** represents the number of the respondents; positive response rate below 50% represents low average positive response rate; 50% – 69.9% represents moderate average positive response rate and 70% and above represents high average positive response rate.

Among the professional nurses, enrolled nurses and enrolled nursing assistants, ‘Teamwork’ (85.48%) and ‘Communication about error’ (80.32%) were also rated as high. In contrast, the operational managers of nursing, assistant managers of nursing and quality assurance managers rated these same composites at moderate and low levels, with scores of 62.84% and 19.92%, respectively.

For the composite ‘Staffing and work pace’, the professional nurses, enrolled nurses and enrolled nursing assistants reported a moderate average positive response rate of 60.03%, while the operational managers of nursing, assistant managers of nursing and quality assurance managers rated it lower at 44.83%, reflecting a low average positive response.

The composite ‘Communication openness’ received a moderate rating of 55.37% from the professional nurses, enrolled nurses and enrolled nursing assistants but a low rating of 4.89% from the operational managers of nursing, assistant managers of nursing and quality assurance managers.

In relation to ‘Organisational learning – continuous improvement’, the professional nurses, enrolled nurses and enrolled nursing assistants provided a low average positive response rate of 43.75%, while the operational managers of nursing, assistant managers of nursing and quality assurance managers rated it moderately at 57.86%.

Both groups reported similarly low ratings – ranging from 10.17% to 32.18% – for the following composites: ‘Reporting patient safety incidents’, ‘Overall perceptions of patient safety’, ‘Response to error’ and ‘Hospital management support for patient safety’.

Overall, the patient safety culture score was moderate for the professional nurses, enrolled nurses and enrolled nursing assistants, at 53.47%, and low for the operational managers of nursing, assistant managers of nursing and quality assurance managers at 40.10%.

Table 3 represents comparable findings for the three hospitals on the composites of patient safety culture.

**TABLE 3:** Comparable findings for the three hospitals on the composites of patient safety culture.

Composite	Percentage				*p*-value
	Central hospital composite positive response rate (*N* = 206)	Regional hospital composite positive response rate (*N* = 128)	District hospital composite positive response rate (*N* = 102)	Average positive response rate for the three hospitals (*N* = 436)	
1. Supervisor, manager or clinical leader support for patient safety	89.56	85.86	89.03	88.15	0.038
2. Handoffs and information exchange	82.99	83.32	85.30	83.87	0.072
3. Teamwork	81.35	80.65	80.43	80.81	0.142
4. Communication about error	64.09	69.80	70.93	68.27	0.454
5. Staffing and work pace	55.00	53.75	62.25	57	0.011
6. Organisational learning – continuous improvement	52.20	38.84	48.64	46.56	0.124
7. Communication openness	33.98	38.73	41.17	37.96	0.027
8. Hospital management support for patient safety	30.01	28.87	33.31	30.73	0.042
9. Response to error	23.15	28	28.86	26.67	0.090
10. Reporting patient safety incidents	22.99	30.73	33.68	29.13	0.041
11. Overall perceptions of patient safety	14.52	15.15	14.37	14.68	0.000
**Overall level of patient safety culture**	49.99	50.34	53.45	51.26	-

*Source*: Adapted from: Famolaro, T., Yount, N.D., Burns, W., Flashner, E., Liu, H. & Sorra, J., 2016, *Hospital survey on patient safety culture: 2016 user comparative database report*, AHRQ Publication No. 16-0021-EF, Agency for Healthcare Research and Quality, Rockville, MD

Notes: *N* represents the number of the respondents; positive response rate below 50% represents low average positive response rate; 50% – 69.9% represents moderate average positive response rate and 70% and above represents high average positive response rate. *P*-value (*p* < 0.05 significance level).

Table 3 presents a comparison of patient safety culture composite scores across the three hospitals. The composites ‘Supervisor, manager or clinical leader support for patient safety’, ‘Handoffs and information exchange’ and ‘Teamwork’ received high average positive response rates ranging from 80.43% to 89.56% across all facilities. The composite ‘Communication about error’ was rated highest at the district hospital (70.93%), while the central and regional hospitals reported moderate scores of 64.09% and 69.80%, respectively.

All three hospitals rated ‘Staffing and work pace’ within the moderate range, with scores between 53.75% and 62.25%. For ‘Organisational learning – continuous improvement’, the central hospital reported a moderate score of 52.20%, while the regional and district hospitals recorded lower scores of 38.48% and 48.64%, respectively.

Four composites – ‘Hospital management support for patient safety’, ‘Response to error’, ‘Reporting patient safety incidents’ and ‘Overall perceptions of patient safety’ – received low average positive response rates across all hospitals, ranging from 14.37% to 41.17%.

The overall average patient safety culture score across the three hospitals was 51.26%, indicating a moderate level of positive perception among healthcare staff.

Statistical analysis revealed that six patient safety culture composites showed statistically significant differences across the facilities. These included Supervisor, manager or clinical leader support for patient safety (*p* = 0.038), Staffing and work pace (*p* = 0.011), Communication openness (*p* = 0.027), Hospital management support for patient safety (*p* = 0.042), Reporting patient safety incidents (*p* = 0.041) and Overall perceptions of patient safety (*p* = 0.000). These variations may be attributed to differences in leadership style, staff training and experience, staffing levels, availability of resources, institutional safety policies and contextual characteristics such as hospital size, location and patient load.

Conversely, five composites did not differ significantly across the three hospitals, suggesting shared patterns in staff perception. These were Handoffs and information exchange (*p* = 0.072), Teamwork (*p* = 0.142), Communication about error (*p* = 0.454), Organisational learning – continuous improvement (*p* = 0.124) and Response to Error (*p* = 0.090). The consistency in these areas may reflect common organisational cultures and systemic challenges across the hospitals, including constrained resources, high workloads and limited training in patient safety practices.

## Discussion

### Key findings

The highest average positive response rates were recorded in three patient safety culture composites: Supervisor, manager or clinical leader support for patient safety, Handoffs and information exchange and Teamwork, indicating relative strengths in these areas across the three hospitals. Moderate scores were observed for ‘Staffing and work pace’ and ‘Reporting patient safety incidents’.

Five composites were identified as areas requiring significant improvement: ‘Organisational learning – continuous improvement’, ‘Communication openness’, ‘Hospital management support for patient safety’, ‘Response to error’ and ‘Overall perceptions of patient safety’.

The overall patient safety culture score across the three hospitals was 51.26%, reflecting a moderate average positive response rate and highlighting the need for targeted interventions to strengthen safety culture more broadly.

### Discussion of key findings

The composites with the highest average positive response rate were ‘Supervisor, manager or clinical leader support for patient safety (88.15%)’, followed by ‘Handoffs and information exchange (83.87%)’ and ‘Teamwork (80.81%)’. Similar results were reported by Osarenmwinda and Akpoavoere (2024), who found a 90.0% rating for ‘supervisor and/or manager expectations and actions promoting patient safety’. These high scores may reflect the influence of strong leadership training and a clear understanding of patient safety responsibilities among clinical leaders. Likewise, Liu et al. (2024) reported an 83% rating for ‘handoffs and information exchange’ in a study conducted in China, which may be attributed to the consistent application of standardised procedures during shift transitions. Sari, Nurharianti and Asih (2025) found teamwork to be rated at 86% in their study at Rumah Sakit Mata Undaan in Surabaya, suggesting that a culture of collaboration and mutual support among healthcare professionals contributes positively to perceptions of teamwork.

Three composites achieved moderate average positive response rates: ‘Communication about error (68.27%)’, ‘Staffing and work pace (57%)’ and ‘Reporting patient safety incidents (54.59%)’. These findings align with those of Al-Zadjali et al. (2025), who reported a 66.7% rating for communication about error among healthcare professionals in Oman. Challenges such as fear of blame, lack of feedback on reported incidents and hierarchical barriers may inhibit open communication. Staffing and work pace also received a 57% rating in the study by Sari et al. (2025), pointing to issues such as staff shortages, high patient volumes, inadequate budgeting and the absence of clear staffing norms. The composite of ‘reporting patient safety incidents’ was rated at 59.5% in a study by Bagnasco et al. (2025). This reluctance to report might be linked to fear of retribution, underdeveloped reporting systems and insufficient follow-up on submitted reports.

The lowest-rated composites in this study were as follows: ‘Organisational learning – continuous improvement (46.56%)’, ‘Communication openness (37.96%)’, ‘Hospital management support for patient safety (30.73%)’, ‘Response to error (26.67%)’ and ‘Overall perceptions of patient safety (14.68%)’. A comparative study by Nwosu et al. (2022) reported a similar result of 45% for organisational learning. Barriers such as limited funding for training and equipment and staffing shortages may prevent meaningful investment in continuous improvement initiatives. Silva et al. (2021) reported a score of 40% for communication openness in a tertiary hospital in Southern Brazil. Factors such as professional hierarchies, lack of confidence among junior staff and inadequate communication training might be the possible contributors to the low scoring of communication openness. Mohammed, Taddele and Gualu (2021) reported a 33.94% score for ‘hospital management support’ in Ethiopia. The low scoring of hospital management support for patient safety might be attributed to limited leadership commitment and inadequate financial and human resources. A study by Alaqeli and Altarhuni (2021) in Benghazi found a 21.06% rating for non-punitive response to error. A culture of blame and a lack of proper training might have contributed to reluctance in error disclosure. Similarly, Afework et al. (2023) reported a 35% rating for ‘overall perceptions of patient safety’. Challenges such as weak trust, ineffective safety protocols and inadequate staff development might be the contributing factors to low ratings for overall perceptions of patient safety.

The overall average positive response rate for patient safety culture across the three hospitals was 51.26%, indicating a moderate level. This is consistent with findings from a study in regional public hospitals in Addis Ababa, which reported an overall rating of 53.72% (Yayehrad, Getachew & Muluken 2024). Factors contributing to these modest ratings may include insufficient leadership involvement, ineffective communication strategies, limited resources, lack of structured safety training, high workloads, resistance to change and a prevailing culture of blame.

### Strengths and limitations

#### Strengths

The use of a descriptive quantitative approach reduced the potential for individual researcher bias by relying on structured numerical data. This method also enhances the reliability and replicability of the study, making it suitable for application in different institutional or geographic contexts.

#### Limitations

The study was limited to three purposively selected public hospitals within Gauteng province, and as such, the results may not be representative of all public healthcare facilities in the province or across South Africa. The findings are also limited to the nurses and quality assurance managers who participated in the current study.

### Recommendations

To strengthen the overall patient safety culture, the three hospitals should prioritise the mobilisation of resources to address staffing shortages and ensure adequate human capacity. In parallel, the development and enforcement of clear policies that encourage non-punitive responses to errors, promote transparent communication and support consistent reporting of patient safety incidents are essential.

Active and visible management support is critical in driving patient safety initiatives. Efforts to foster continuous improvement should include mechanisms for open dialogue about errors and learning from patient safety incidents to prevent recurrence.

It is also important to investigate the underlying factors contributing to the negative perceptions of patient safety among nursing staff and quality assurance managers. Identifying these causes will support the implementation of targeted interventions aimed at improving the safety climate within public hospitals.

## Conclusion

This study assessed the level of patient safety culture within public hospitals, uncovering a moderate overall positive response rate of 51.26%. Key strengths were identified in the areas of ‘supervisor, manager, or clinical leader support for patient safety’, ‘handoffs and information exchange’ and ‘teamwork’. However, ‘organisational learning – continuous improvement’, ‘communication openness’, ‘hospital management support’, ‘response to error’ and ‘overall perceptions of patient safety’ – emerged as areas requiring focused improvement.

The recommendations offered aim to address these gaps by fostering a culture of continuous learning, strengthening leadership visibility and encouraging open communication. These findings provide valuable insights for policymakers, healthcare practitioners and researchers seeking to enhance patient safety strategies and develop targeted interventions.

Ultimately, this study contributes to a broader understanding of patient safety culture within the South African public healthcare context. By implementing the proposed strategies, healthcare institutions can advance safety culture, reduce the occurrence of patient safety incidents and improve patient outcomes.
